# Clinical Features, Radiological Findings, and Treatment Outcomes in Patients with Pulmonary Nocardiosis: A Retrospective Analysis

**DOI:** 10.7759/cureus.17250

**Published:** 2021-08-17

**Authors:** Prakrati Yadav, Deepak Kumar, Durga Shankar Meena, Gopal K Bohra, Vidhi Jain, Pawan Garg, Naveen Dutt, Kumar S Abhishek, Ashwini Agarwal, Mahendra K Garg

**Affiliations:** 1 Medicine, All India Institute of Medical Sciences, Jodhpur, IND; 2 Microbiology, All India Institute of Medical Sciences, Jodhpur, IND; 3 Diagnostic and Interventional Radiology, All India Institute of Medical Sciences, Jodhpur, IND; 4 Pulmonary and Critical Care Medicine, All India Institute of Medical Sciences, Jodhpur, IND

**Keywords:** pulmonary nocardiosis, cell mediate immunity, modified zn staining, trimethoprim sulfamethoxazole, computer tomography of chest

## Abstract

Introduction

Lack of specific clinical features makes the diagnosis of pulmonary nocardiosis difficult. A high index of suspicion is required for diagnosis especially in cohorts with pre-existing risk factors. This study aimed to study the clinical and radiological characteristics and outcomes in patients with pulmonary nocardiosis.

Methods

This was a retrospective observational study. Data of confirmed cases with pulmonary nocardiosis were collected from a digital patient management system.

Results

A total of eight cases of pulmonary nocardiosis were included. The mean age of patients was 50 ± 14.3 years with a female preponderance (62.5%). The most common co-morbidity was chronic lung disease (37.5%). The common clinical feature of pulmonary nocardiosis was cough with expectoration (50%) and the mean duration of symptoms was 18 days. The common radiological (CT thorax) findings were consolidation, bronchiectasis, mediastinal lymphadenopathy, and nodularity (50% each). One patient had an extension of pulmonary disease in the chest wall. Microbiological detection of Nocardia spp. was done in sputum samples (50%) and in bronchoalveolar lavage (BAL) samples (50%). Culture was positive in two BAL samples. Intravenous empirical antibiotics in combination with oral trimethoprim-sulfamethoxazole double standard (15 mg/kg trimethoprim) were started at the time of diagnosis. Ceftriaxone and amikacin were commonly used antimicrobials.

Conclusion

*Nocardia spp.* commonly causes disease in patients with pre-existing chronic disease. A high index of suspicion is required in patients with subacute to chronic respiratory symptoms, raised inflammatory markers, and the absence of common respiratory pathogens in evaluation.

## Introduction

Pulmonary nocardiosis is infrequently diagnosed but is common in patients with pre-existing lung disease with or without defective cell-mediated immunity [1]. Lack of specific clinical features and awareness in community physicians further delays the diagnosis of pulmonary nocardiosis. Nocardia is a ubiquitous organism, and due to abundance in the environment, the burden of Nocardiosis would be higher compared to reported in the literature. Pulmonary Nocardiosis is usually not entertained as an initial diagnosis and is often missed. It has a high mortality, and early diagnosis of disease would be helpful in improving survival [2]. Common portals of entires are inhalational (lung infection), traumatic inoculation (skin ), and contaminated medical equipment (hospital-acquired). Diabetes, malignancies, HIV infections, alcoholism, long-term corticosteroids, etc. are common risk factors of pulmonary nocardiosis. A high index of suspicion is required for diagnosis, especially in a cohort with defective cell-mediated immunity where other common organisms (like tuberculosis and fungus) have been ruled out. This study reports retrospective data of eight cases of pulmonary nocardiosis with a focus on clinical features, associated lung conditions, risk factors, radiological findings, and outcome of treatment.

This article was previously posted to the Research Square preprint server on April 23, 2021.

## Materials and methods

This was a retrospective observational study conducted in a tertiary care center in the western part of India. The study period was March 2019 to March 2020. Data of confirmed cases with pulmonary nocardiosis were collected from the digital patient management system.

The diagnosis of pulmonary nocardiosis was considered when the organism was isolated from the respiratory samples (at least in two sputum samples and/or at least in one bronchoalveolar lavage) and negative for other microbial organisms that could be responsible for the infection.

Data collection included data on demography, clinical symptoms, concurrent diseases, prior corticosteroid and immunosuppressive therapy, laboratory and radiological findings, dissemination to other organs, and outcome of antibiotic therapy.

Microbiological identification of Nocardia spp. was done under standard laboratory guidelines, including direct microscopy and culture. Direct microscopy of sputum sample with modified Ziehl-Neelsen (ZN) staining (1% H_2_SO_4_, Kinyoun method) showed filamentous branching, and beaded acid-fast bacilli were considered Nocardia spp. The culture of the sputum sample was incubated on 5% sheep blood agar and Mac Conkey’s agar for two to seven days at 35-36°C, and identification of colonies was made on the basis of their phenotypic, metabolic, biochemical, and growth tests on different temperatures. Interpretations of culture results were noted 24, 48, and 72 hours after the incubation. Common pulmonary pathogens (bacterial, fungal, mycobacterium tuberculosis) were sought using Gram stain, ZN stain, sputum culture, and blood cultures. Contrast-enhanced computer tomography (CECT) of the thorax was analyzed for all the patients. Abnormalities on CECT were classified as ground-glass opacity, consolidation, nodules, cavitary lesion, bronchiectasis, and mediastinal lymphadenopathy. The presence and absence of pleural and pericardial effusion were also noted.

## Results

**Figure 1:**
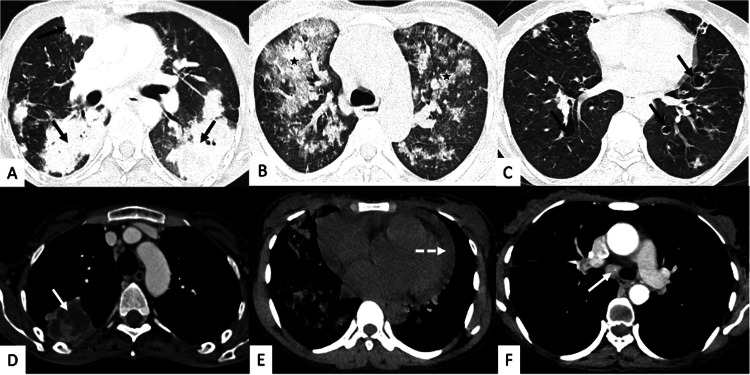
Computed tomography (CT) thorax in different patients Case 1: High-resolution CT (HRCT) (A) and contrast-enhanced CT (CECT) (D) showing a multifocal patchy area of consolidation (black arrows) with internal necrosis (white arrow). Case 2: HRCT (B) and noncontrast CT (NCCT) (E) showing diffuse consolidation and ground glass in bilateral lungs (asterisk) with pericardial effusion (dashed arrow). Case 3: HRCT (C ) and CECT (F) showing bronchiectasis (black arrows) with multiple parenchymal nodules and mediastinal lymphadenopathy (white arrow).

Demographic characteristics and underlying comorbidities are shown in Table 1. The mean age of patients was 50 ± 14.3 years (range 29-72 years) with female preponderance (62.5%). All patients have at least one underlying comorbidity, as depicted in Table 1. The most common comorbidity was chronic lung disease found in three (37.5%) patients (two had chronic obstructive pulmonary disease and one had post-tubercular lung fibrosis). The most typical clinical feature of pulmonary nocardiosis was cough with expectoration, which was present in all patients, and fever was present only in 4 (50%) patients (Table 2). The mean duration of symptoms was 18 days (range, 7-30), and the maximum patients had symptoms for more than 14 days. Two patients were clinically diagnosed with pulmonary tuberculosis and were on anti-tubercular therapy. One patient had a history of pulmonary tuberculosis three years back and received a full six months’ treatment. Anemia (10.67 ± 1.87 in gm%) and leukocytosis (10182 ± 5842 cell/cumm) were present in 42.85% of patients, and inflammatory markers were significantly raised in most of the patients (high-sensitivity C-reactive protein (Hs-CRP) 84.5 ± 73.11 and erythrocyte sedimentation rate (ESR) 53.14 ± 14.67) (Table 2). One patient had associated idiopathic CD4 T cell lymphocytopenia. The absolute CD4 count of this patient was 212 cell/µL and 191 cell/µL on two occasions three months apart and negative for HIV-1 and HIV-2 on both occasions. The radiological (CT thorax) findings are shown in Table 3. Consolidation, bronchiectasis, mediastinal lymphadenopathy, and nodularity were common findings (Figure 1). Involvement of unilateral lung parenchyma was found in four (50%) patients. One patient had invasive disease with extension in the chest wall (Table 3). Microbiological detection of Nocardia spp. was done in two consecutive sputum samples in four patients (50%) and BAL samples in four patients (50%). The culture was positive in two BAL samples. Intravenous empirical antibiotics in combination with oral trimethoprim-sulfamethoxazole double standard (15 mg/kg trimethoprim) were started at the time of diagnosis. Ceftriaxone and amikacin were commonly used antimicrobials (Table 4). One patient (62-year-old male), who had underlying squamous cell carcinoma of buccal mucosa with brain metastasis, died during treatment.

**Table 1:** Demography and underlying risk factors of patients with pulmonary nocardiosis

Characteristic	n (%)
Total Patients	8
Mean age (±SD) (years)	50 ± 14.3
Sex distribution	
Male	3 (37.5%)
Female	5 (62.5%)
Underlying conditions	
Chronic lung disease	3 (37.5%)
Chronic kidney disease	1 (12.5%)
Malignancy	1 (12.5%)
Steroid and immunosuppressive drugs (autoimmune disease)	1 (12.5%)
Pancytopenia	1 (12.5%)
Idiopathic CD4 lymphocytopenia	1 (12.5%)

**Table 2:** Clinical and laboratory features of the patients with pulmonary nocardiosis Hs-CRP: high-sensitivity C-reactive protein; ESR: erythrocyte sedimentation rate

Characteristic	n (%)
Clinical Features	
Fever	4 (50%)
Cough	8 (100%)
Expectoration	8 (100%)
Breathlessness	5 (62.5%)
Weight loss	3 (37.5%)
Laboratory investigations	
Anemia	3 (37.5%)
Leukocytosis	3 (37.5%)
Neutrophilia	1 (12.5%)
Lymphocytosis	1 (12.5%)
High HsCRP	7 (87.5%)
High ESR	8 (100%)

**Table 3:** Findings of computer tomography (CT) thorax in patients with pulmonary nocardiosis

CT Thorax finding	n (%)
Consolidation	3 (50%)
Ground-glass opacity	2 (25%)
Cavitary lesion	3 (37.5%)
Nodules	4 (50%)
Bronchiectasis	4 (50%)
Fibrotic changes	4 (50%)
Mediastinal lymphadenoapthy	4 (50%)
Pleural effusion	1 (12.5%)
Pericardial effusion	1 (12.5%)

**Table 4:** Antibiotic regimens and outcome in patients with pulmonary nocardiosis

Antibiotic combinations	Treated patients/deaths
Ceftriaxone + amikacin + trimethoprim-sulfamethoxazole DS	4/0
Imipenem + amikacin + linezolid	1/1
Amikacin + trimethoprim-sulfamethoxazole DS	3/0

## Discussion

Nocardia is an aerobic, Gram-positive filamentous bacteria that commonly affects the respiratory system in humans. Nocardia can cause disseminated disease involving the central nervous system (CNS) and skin or rarely other organs [3]. It can cause acute, subacute, and chronic disease and a high index of suspicion is required for diagnosis. Pulmonary nocardiosis is considered an opportunistic infection and occurs mainly in patients with defective cellular immunity. Due to a lack of specific clinical features, the diagnosis of pulmonary nocardiosis turns out to be complicated. Most of the time, pulmonary tuberculosis, lung abscess, bronchiectasis with secondary bacterial infection, invasive fungal infection, etc., are initial diagnoses in these patients. This study is focused on clinical and laboratory features in patients with pulmonary nocardiosis to increase awareness among community physicians.

The mean age of patients in this study was 50 ± 14.3 years, and disease was common in females. Contrary to this, previous literature had a higher incidence of pulmonary nocardiosis in males [1,4-8]. This difference may be due to a smaller number of cases or a lack of more extensive studies. Chronic obstructive lung disease (COPD) was the most common risk factor associated with around 40% of cases. COPD and chronic steroid use are the crucial pre-existing factor for pulmonary nocardiosis, and more than half of patients had the above comorbidities in previous studies [1-3,8-12]. Patients with malignancy, leukemia, HIV, diabetes, or cytotoxic chemotherapy and organ transplant recipients also have an increased risk for nocardiosis. Intact cell mediates immunity is crucial for containment of Nocardia infections [10]. Evaluation of cell mediates immunity in the form of CD4 count is helpful in the understanding of pathogenesis in cases of nocardiosis without comorbidities. The association of idiopathic CD4 lymphocytopenia and pulmonary nocardiosis is less studied. In this study, one patient had low CD4 (less than 300 cell/µL) and on two occasions, was negative for HIV 1 and 2. We recommend that CD4 count be done in every case of nocardiosis to find an association with ICL and further prophylaxis.

The disease duration was subacute or chronic in most cases, but one patient, who also had pancytopenia, presented with six days of illness. High suspicion should be kept even in acute presentation, especially in patients with risk factors [3]. The typical clinical features were cough, expectoration, and breathlessness, while fever was seen only in half cases. These clinical features are non-specific and often directed towards tuberculosis or fungal infection. The invasive nature of the disease is an important diagnostic clue, and one patient had an extension of the disease in the chest wall, which was diagnosed in the CECT thorax. Anemia, lymphocytosis, neutrophilia, and raised inflammatory markers were similar to other cases series [1,12]. Persistent symptoms, raised inflammatory markers and lack of other infective etiology in patients with chronic lung disease become a high index of suspicion for pulmonary nocardiosis.

Pleural effusion and consolidation were common radiological findings in pulmonary nocardiosis [11]. Liu B et al. reported chest CT findings of nine cases [13]. Common CT thorax findings in pulmonary nocardiosis were parenchymal nodules, consolidation, mediastinal lymphadenopathy, with or without cavitation present in around 70% of patients. Above CT abnormality in immunocompromised patients with high clinical suspicion may suggest the likelihood of pulmonary nocardiosis [13]. Confirmation of diagnosis required the identification and isolation of Nocardia spp. In modified acid‑fast staining using 1% sulfuric acid and/or culture in sputum or BAL sample [1]. In this study, all patients had positive for modified acid‑fast staining, showed filamentous branching bacilli consisted with Nocardia spp. BAL was done in four cases, and out of them, two had a positive culture for Nocardia spp. Species identification was not made due to resource constrain. A clinical background should be taken care of during isolation of Nocardia spp. to avoid overdiagnosis and superfluous use of antibiotics.

There is no definitive treatment guideline for nocardiosis, and most of the data are from retrospective studies. Combination antibiotic therapy with trimethoprim‑sulfamethoxazole (25-50 mg/kg per day of sulfamethoxazole) is the most widely used in treatment [1,3]. Amikacin, ceftriaxone, imipenem, and linezolid have been showing effectiveness in various combinations. Pulmonary disease with moderate symptoms (50% cases) were treated with a combination of ceftriaxone plus amikacin plus Trimethoprim‑sulfamethoxazole showed an effective cure in this study. Patients should be treated for at least six months or until the resolution of disease, especially in immunocompromised patients [14]. Mortality is high in nocardiosis, and one long-term study showed 41%, 64%, and 100% mortality in pulmonary, disseminated, and central nervous system (CNS) disease, respectively [2]. However, a recent study from Australia found all-cause mortality in nocardiosis was 15% and 22% at six and 12 months [11]. Similarly, this study also had morality in 12.5% of cases at six months.

Limited studies have been reported from India regarding pulmonary nocardiosis, and most of them are case reports [1,3,8,15-19]. From the western part of India, these only a few cases have been reported [20-21]. This study primarily focuses on clinical and radiological features of pulmonary nocardiosis to increase awareness in community clinicians.

The limitations of the study were the retrospective analysis and species detection, as well as drug sensitivity testing, which couldn’t be possible due to resource constraints.

## Conclusions

Nocardia spp. commonly causes disease in patients with pre-existing lung pathological with or without defective cell-mediated immunity. The typical clinical features are cough, expectoration, and breathlessness. Most patients had subacute disease duration. Common radiological findings in pulmonary nocardiosis are parenchymal nodules, consolidation, and mediastinal lymphadenopathy with or without cavitation. A high index of suspicion is required in patients with subacute to chronic respiratory symptoms, raised inflammatory markers, and absence of common respiratory pathogens in evaluation.
